# Functional Characterization of *FH* Mutation c.557G>A Underlies Uterine Leiomyomas

**DOI:** 10.3390/ijms23031452

**Published:** 2022-01-27

**Authors:** Ping Li, Yanru Wu, Huizhi Wu, Qiuhong Xiong, Na Zhao, Guangxin Chen, Changxin Wu, Han Xiao

**Affiliations:** Institutes of Biomedical Sciences, The Key Laboratory of Chemical Biology and Molecular Engineering of Ministry of Education of China, The Key Laboratory of Medical Molecular Cell Biology of Shanxi Province, Shanxi University, Taiyuan 030006, China; 18434756025@163.com (Y.W.); wuhuizhi1994@163.com (H.W.); qxiong@sxu.edu.cn (Q.X.); zyzdxy@sina.com (N.Z.); chengx@sxu.edu.cn (G.C.); cxw20@sxu.edu.cn (C.W.)

**Keywords:** uterine leiomyomas, FH, fumarase, mutation, mTOR, autophagy

## Abstract

The *FH* gene encodes the fumarate hydratase of the Krebs cycle and functions as a homotetramer to catalyze the hydration of fumarate to malate. Mutations in *FH* result in uterine leiomyomas, a rare autosomal dominant inherited metabolic disease. However, how *FH* mutations result in this disease is poorly understood. Here, the *FH* mutation c.557G>A (p.S186N) was identified in a family with uterine leiomyomas phenotype. A series of studies were performed to confirm the pathogenicity of this mutation. Results showed that the FH mutant exhibited significantly lower fumarase enzyme activity and increased the fumarates level compared with the wildtype, which might be due to the impaired homotetramer formation in the native gel electrophoresis. Interestingly, the immunofluorescence study revealed that the overexpressed FH mutant exhibited puncta structures compared with the evenly expressed FH wildtype in cytoplasm suggesting that the altered amino acid might result in dysfunctional proteins which were accumulated to reduce its cytotoxicity. Importantly, the cells overexpressing the FH mutant exhibited higher proliferation and extracellular acidification rate value (ECAR) which might be caused by the upregulated HIF-1α indicating the tumor phenotype. Notably, phospho-mTOR was significantly increased and autophagy was inhibited in the FH mutant overexpression cells compared with the wildtype. Our work provides new insight into the *FH* mutation c.557G>A (p.S186N) underlies uterine leiomyomas and important information for accurate genetic counseling and clinical diagnosis of the disease.

## 1. Introduction

Uterine leiomyomas (ULMs), together with multiple cutaneous (CLMs) and renal cell carcinomas (RCCs), are known as hereditary leiomyomatosis and renal cell cancer (HLRCC), a rare autosomal dominant inherited metabolic disease, resulting from fumarate hydratase (FH, NG_012338.1) gene mutations on chromosome 1q42.3-43 [1], which lead to multiple cutaneous leiomyomas, uterine leiomyomas (in women) and renal cell carcinomas.

Multiple cutaneous (CLMs) occurring as smooth-surfaced, erythematous hyperpigmented or skin-colored, solitary or multiple papules or nodules are often the basic representation and typical diagnosis marker of the disease [2,3]. Uterine leiomyomas (ULMs) are highly penetrant as 73% to 100% of women with the *FH* mutation are affected by the common symptoms including menorrhagia, irregular menstruation, pain and reproductive dysfunction which often lead to infertility and pregnancy complications [4,5]. Renal cell carcinomas (RCCs) had been reported to be associated with skin and uterine leiomyomas in 2001 [6]. Although most patients with RCCs are asymptomatic, the disease is still invasive since the majority of patients died of metastatic disease in a short period after diagnosis [7].

*FH* encodes the fumarase enzyme which converts fumarate to malate in the tricarboxylic acids (TCA; Krebs) cycle contributing to cellular energy metabolism. It is evolutionarily highly conserved from human to other eukaryotic cells and exhibits enzyme activity as a homotetramer, formed by four identical subunits with 50 kDa [8]. FH was identified as a tumor suppressor due to the lower enzyme activity or deficiency in tumors from patients with HLRCC [9]. More and more studies suggest that the reason for tumorigenesis in fumarase-deficient cells is due to fumarate accumulation leading to upregulation of HIF in the cytoplasm [10,11].

To date, more than one hundred *FH* causative mutations have been reported in HGMD (Human Gene Mutation Database, http://www.hgmd.org/, 1 January 2022). However, most of the *FH* variants were only reported as clinical reports without functional validation. In this study, the *FH* missense mutation c.557G>A (p.S186N) was identified from a Chinese female patient with uterine leiomyomas. Although this alteration has been detected with an allele frequency of approximately 0.02% (greater than 10,000 alleles tested) in Ambry Genetics’s clinical cohort in the ClinVar database, there is no experimental evidence for its pathogenicity. The aim of this study was to demonstrate the molecular mechanism of the c.557G>A (p.S186N) mutation underlies uterine leiomyomas.

## 2. Results

### 2.1. Patient Information and Sequencing Results

The patient has regular menstruation (5/30 days) in moderate volume without dysmenorrhea. The B-ultrasonic examination showed that the uterine leiomyoma (4cm in diameter) was rich in blood flow. Considering the risk of myoma degeneration, the hysteromyomectomy was performed and postoperative pathological (biopsy) examination showed that: (uterus) cells were significantly heteromorphic without obvious necrosis and mitosis. The patient was diagnosed as atypical uterine leiomyoma.

**Figure 1:**
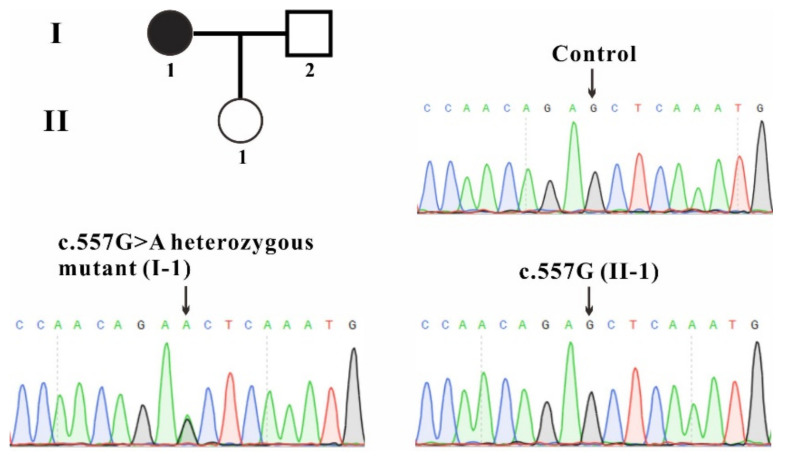
Family pedigree and sequencing results. The patient involved in this study is shown as filled circles. Sanger sequencing results from the patient (I-1), her daughter (II-1) and a healthy control were shown and the gene variation is indicated by a black arrow.

To identify whether or which gene mutation is disease-causative in this family, the blood samples from the patients and one healthy volunteer were collected and whole-exome sequencing was performed. The *FH* mutation c.557G>A (p.S186N) was identified to be the probable disease-causing mutation. Gene co-segregation was confirmed by Sanger sequencing for individuals I-1 and II-1 in this pedigree (Figure 1) and heterozygous single base substitution in exon 5 of *FH* (c.557G>A, p.S186N) was identified only in patient I-1 and this mutation was predicted to be disease-causing by Mutation Taster (disease-causing, score: 0.999), PolyPhen-2 (possibly damaging, score: 0.523) and PROVEAN (deleterious, score: 2.951).

### 2.2. FH c.557G>A Mutation Resulted in Reduced Enzyme Activity and Increased Cellular Fumarates

**Figure 2:**
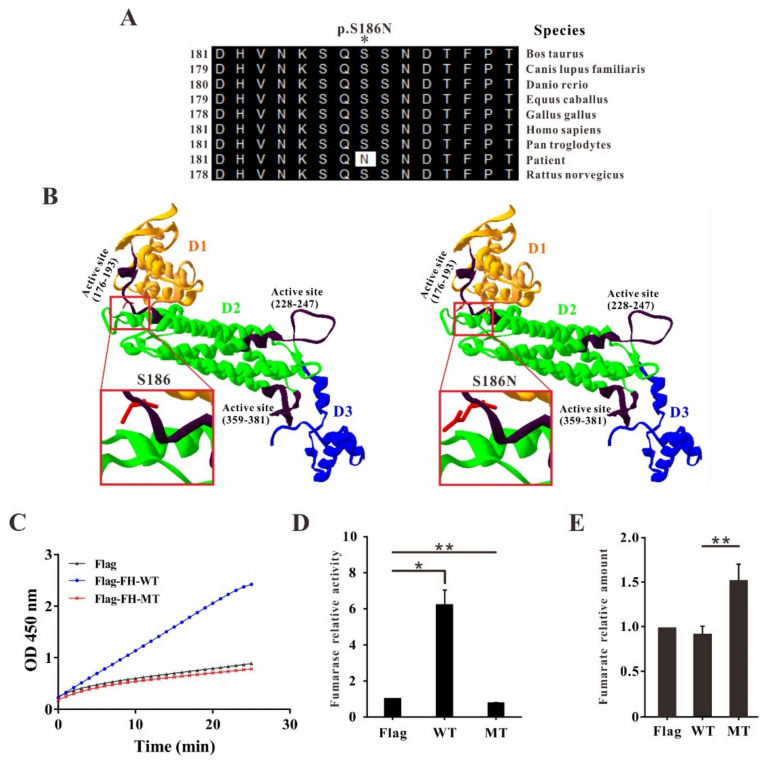
Analysis of *FH* mutation. (**A**) Evolutionary conservation of amino acid residues altered by c.557G>A (p.S186N) across different species. NCBI accession numbers are: Bos Taurus: NP_001069271.1; Canis lupus familiaris: XP_537215.1; Danio rerio: NP_957257.1; Equus caballus: XP_023488568.1; Gallus gallus: NP_001006382.1; Homo sapiens: NP_000134.2; Pan troglodytes: NP_001267172.1; Rattus norvegicus: NP_058701.2. (**B**) The mutant protein was structured by Swiss-Model online software compared to the wildtype. Three domains of FH monomer are shown with different colors, D1-yellow, D2-green and D3-blue. Three active sites from each monomer are presented in black. Amino acid S186 and S186N are shown as red sticks. (**C**) The absorbances were measured at 450 nm in kinetic mode with Flag-FH wildtype or mutant stable overexpression HEK293T cells. (**D**) Relative fumarase activity was calculated in corresponding cells. (**E**) Cellular fumarates level was significantly increased in FH mutant overexpression cells (* *p* < 0.05, ** *p* < 0.01). All data are presented as the mean ± SD from at least three separate experiments.

To date, more than one hundred *FH* gene mutations have been reported and most of them are missense mutations [12]. To identify the evolutionary conservation of the altered amino acid, multiple sequence alignment was performed. The result showed that impaired amino acid residue S186 was highly evolutionary conserved among FH proteins from different species, indicating this mutation was likely to be the causative mutation resulting in uterine leiomyomas (Figure 2A).

The crystal structure of the FH recombinant enzyme revealed that the human FH structure corresponds to a homotetramer protein [8]. Each monomer consists of three domains termed as D1 (residues:49–188), D2 (residues: 189–439) and D3 (residues: 440–510). D2, the center domain, functions in subunit interaction, namely tetramerization. The D1 and D3 domains are located at the corners of the homotetramer and format the entrance to the independent four fumarase active sites. Each active site is composed of three regions, region 1 (residues 176–193), region 2 (residues 228–247) and region 3 (residues 359–381) from different monomers [8,13,14]. Molecular modeling showed that the altered amino acid residue S186 was involved in the active site of region 1 suggesting that the enzyme activity might be affected (Figure 2B).

HEK293T cells are widely used in many kinds of studies including signal transduction and protein interaction studies over viral packaging to rapid small-scale protein expression and biopharmaceutical production [15]. To test our hypothesis, the Flag-FH wildtype or mutant stable overexpression HEK293T cells were constructed by means of lentiviral transfections and the fumarase activities were measured with these cell lines. The absorbances were measured and the activities were calculated. In wildtype FH overexpression cells, the absorbance was rapidly increased up to ~2.5 within 25 min at 450 nm reflecting the highly enhanced enzyme activity (Figure 2C). Interestingly, the absorbances of both Flag only and FH mutant overexpression cells were slowly increased up to ~0.89 and ~0.78, respectively (Figure 2D). In Flag only overexpression control cells, the increased absorbance revealed the enzyme activity of endogenous fumarase. Most strikingly, in Flag-FH mutant overexpression cells, the increased absorbance was similar and even lower than that from the control cells (Figure 2D) and the fumarates level was significantly increased in Flag-FH mutant overexpression cells consistently (Figure 2E), indicating the enzyme activity of FH mutant is impaired which could even affect the endogenous fumarase.

### 2.3. Abnormalities of Fumarase Homotetramer and Localization in FH Mutant Transfected Cells

**Figure 3:**
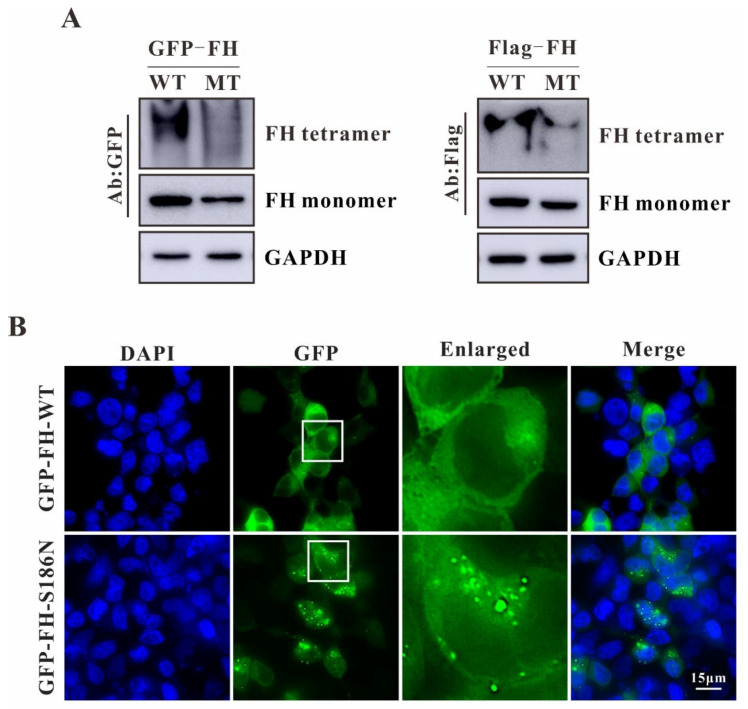
Abnormalities of fumarase homotetramer and localization with FH mutant. (**A**) Native gel electrophoresis was performed to analyze the FH tetramer formed in HEK293T cells expressing either flag tagged or GFP tagged FH wildtype and mutant, respectively. All data are presented from at least three separate experiments. (**B**) Immunofluorescence staining was performed in HEK293T cells transfected with GFP-FH-WT or GFP-FH-MT plasmids. Bar: 15 μm.

It was known that from human to other eukaryotic cells, fumarases are evolutionarily highly conserved homotetrameric enzymes with a molecular weight about 200 kDa [8]. Some reports suggest that the fumarase exhibits enzyme activity only as a tetramer [16,17]. Therefore, we ask whether this mutation affects the FH tetramerization. To answer this question, native gel electrophoresis was performed. We analyzed the FH tetramers formed in HEK293T cells expressing either the flag tagged or the GFP tagged FH wildtype and mutant, respectively. The results from the native gel showed that both the GFP-tagged and Flag-tagged FH wildtype could form the homotetramer while the formation was impaired in HEK293T cells expressing FH mutant suggesting that the impaired tetramer formation might be the reason for the reduced enzyme activity since each active site contains three regions from different monomer (Figure 3A).

To identify whether this mutation affects the localization of FH inside the mutant transfected cells, we transiently transfected the GFP-FH wildtype or mutant into HEK293T cells. In GFP-FH-WT transfected cells, the FH fusions were evenly expressed in the cytoplasm. Surprisingly, puncta structures were exhibited in the cytoplasm of GFP-FH-MT transfected cells indicating that the altered amino acid might affect the correct folding of the protein (Figure 3B).

### 2.4. Dysregulation in mTOR Signaling and Autophagy

Uterine leiomyomas are characterized by the mutation from fumarate hydratase, one of the enzymes in the Krebs cycle, which catalyzes the hydration of fumarate to malate [3]. The contributions of the *FH* mutations to the disease are unknown. Therefore, we carried out investigations on the cellular level to probe for alterations that are associated with the disease.

**Figure 4:**
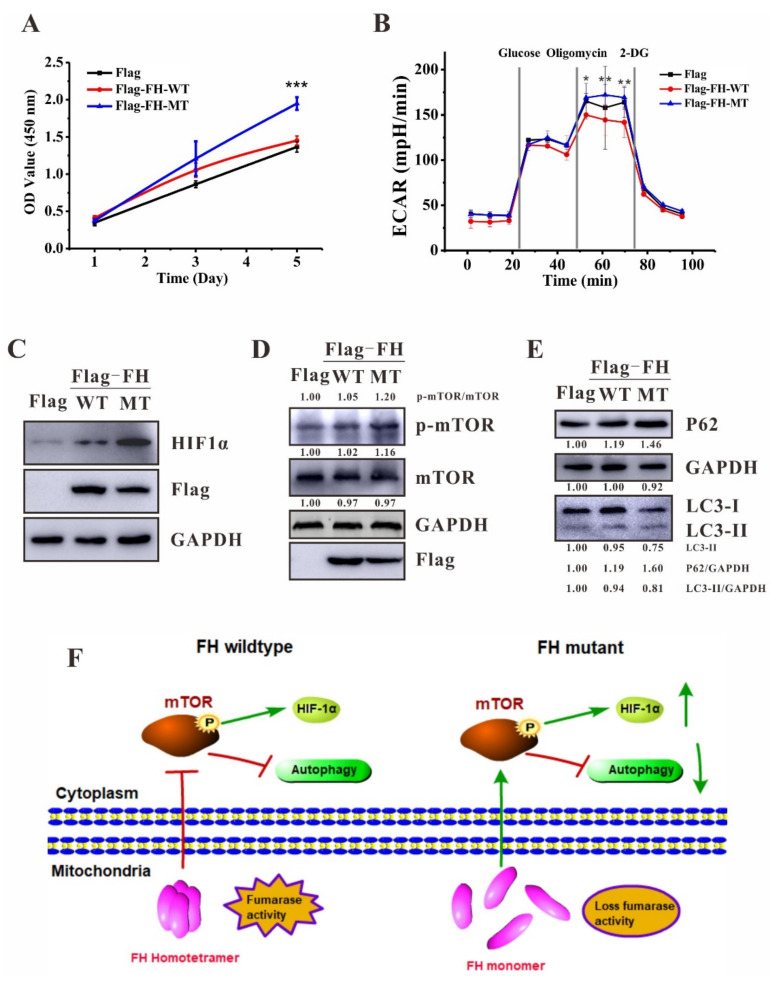
Dysregulation in mTOR signaling and Autophagy. (**A**) Cell proliferation of Flag only, Flag-FH wildtype or Flag-FH mutant overexpression HEK293T cells. The significance between Flag-FH-WT and Flag-FH-MT was indicated (*** *p* < 0.001). (**B**) Extracellular acidification rate (ECAR) was measured after consecutive injections of glucose (10 mM), oligomycin (1 μM), and 2-DG (50 mM) in stable cell lines. The significances between Flag-FH-WT and Flag-FH-MT were indicated (* *p* < 0.05; ** *p* < 0.01). (**C**) HIF-1α was analyzed by western blotting in stable cell lines upon treatment with MG132 at a final concentration of 10 nM for overnight. (**D**) Western blotting for mTOR activation on stable cell lines. (**E**)The expressions of LC3-II and p62 were measured in stable cell lines. GAPDH was used as an internal loading control. All data are presented as the mean ± SD from at least three separate experiments. (**F**) Proposed mechanism of *FH* mutation results in uterine leiomyomas. In the presence of FH wildtype, fumarase homotetramer is formed to exhibit the enzyme activity, resulting in the inhibition of mTOR signaling. In the presence of FH mutant, fumarase homotetramer is impaired and the enzyme activity is lost, resulting in the activation of mTOR, which further leads to the upregulation of HIF-1α and downregulation of autophagy.

To evaluate the cell proliferation ability of cells harboring *FH* mutation, 2 *×* 10^3^ cells of stable overexpression Flag, Flag-FH wildtype or mutant were seeded in a 96-well plate and counted every 48 h for 4 days. The cells stabling overexpression FH wildtype exhibited similar growth compared to the Flag-only overexpression cells. In contrast, the cells stabling overexpression FH mutant showed significantly increased growth compared to wildtype (Figure 4A).

Glycolysis and oxidative phosphorylation are the two major energy-producing pathways that are linked by the tricarboxylic acid (TCA) cycle under aerobic conditions in the cell. To adapt to their environment, most cells can switch between these two pathways [18,19]. To identify the possible effect of the *FH* mutation on energy producing in mitochondria, the extracellular acidification rate (ECAR) was measured in stable cell lines. Oligomycin, an ATP synthase inhibitor, can inhibit mitochondrial ATP production, and switch the energy production to glycolysis, with the subsequent increase in ECAR revealing the cellular maximum glycolytic capacity. After the injection of oligomycin, the ECAR value of Flag, Flag-FH wildtype or mutant stable overexpression cells were significantly increased (Figure 4B). In Flag-only overexpression control cells, the increase of the ECAR value after oligomycin injection means the drug treatment worked under our experimental condition. In FH wildtype overexpression cells, the increase of the ECAR value (cellular maximum glycolytic capacity) was much less than the control cells revealing that the converted pyruvate from glucose in glycolysis was rapidly subjected to the tricarboxylic acid (TCA) cycle under aerobic conditions indicating the overexpressed FH wildtype was highly activated. Most strikingly, in FH mutant overexpression cells, the increased ECAR value was similar to the control cells suggesting the overexpressed FH mutant was inactive (Figure 4B).

It was reported that FH-deficient human tumors have an increased hypoxia-inducible factor (HIF) activity that contributes to rapid tumor growth and proliferation [18,20,21]. To determine whether the *FH* mutation causes HIF upregulation or not, Flag, Flag-FH wildtype or mutant stable overexpression cells were analyzed by western blotting. HIF-1α was induced by hypoxia and degraded via proteasome pathway [22,23], further, it is undetectable in HEK293T cells under normoxia. Therefore, to verify whether the FH mutant promotes the expression of HIF-1α, proteasome inhibitor MG132 was applied to the cells with a final concentration of 10 nM overnight and then HIF-1α was detected by western blotting. The result revealed that the overexpressed FH mutant causes a significant upregulation of HIF-1α under MG132-mediated proteasomal blocked condition (Figure 4C).

The mammalian target of the rapamycin (mTOR) pathway, which regulates cell growth and metabolism, is often activated in cancer and plays an important role in oncogenesis [24]. Moreover, mTOR is identified as an upstream activator of HIF-1α functions in cancer cells [25]. Thus, we wonder whether the upregulated HIF-1α was due to the activation of mTOR in our cell model. To answer this question, western blotting was performed with Flag, Flag-FH wildtype or mutant stable overexpression cells. As shown in Figure 4D, the overexpression of the FH mutant significantly increased the expression level of phospho-mTOR compared to the control and FH wildtype transfected cells indicating that the dysfunction of FH could activate mTOR.

Further, the activation of mTOR signaling would downregulate the autophagy and suppress the formation of autophagic vesicles [26]. Therefore, we tested whether the *FH* mutation could alter the activity of autophagy pathways in our stable overexpression cells. Interestingly, the FH mutant overexpression cells showed a remarkable downregulation of autophagic marker LC3- II and upregulation of p62, which is a substrate of autophagy, indicating autophagy is inhibited upon the *FH* mutation (Figure 4E). 

## 3. Discussion

In this study, a 29-year-old female patient was recruited 48 days after myomectomy and diagnosed as uterine leiomyoma in China. To find out whether this disease is a genetic disorder, whole-exome sequencing was performed and a heterozygous single base substitution in exon 5 of *FH* (c.557G>A, p.S186N) was identified in the patient (Figure 1). Considering that the daughter of the patient does not carry this mutation and the genotypes of her parents are not available, we speculate that this mutation might be inherited from one of her parents or it is a de novo germline mutation.

The *FH* gene encodes the fumarate hydratase enzyme on chromosome 1q42.3–q43 which belongs to the TCA cycle (Krebs cycle) converting fumarate to malate. The germline heterozygous mutation of the *FH* gene results in HLRCC including multiple cutaneous, uterine leiomyomas and renal cell carcinomas. For female patients, uterine leiomyomas occur in almost all women with an average age of thirty years old and lead to dysmenorrhea, menstrual irregularities, menorrhagia and even infertility, suggesting the importance of early diagnosis [27].

Although this variation is a missense mutation without protein length alteration, the fumarase enzyme activity was highly affected. Sequence analysis showed that the impaired amino acid serine186 was highly conserved among FH proteins from multiple species suggesting the great importance of this amino acid (Figure 2A). The crystal structure of FH is a homotetramer containing four independent fumarase active sites and each active site is composed of three regions, region 1 (residues 176–193), region 2 (residues 228–247), region 3 (residues 359–381) from different monomers [13,14]. Molecular modeling revealed that the altered amino acid residues S186 were located in the active site of region 1 suggesting the inactivation of the enzyme with this variation (Figure 2B). Our data showed that the cells stable overexpressing of FH mutant exhibited significantly lower fumarase enzyme activity which is even lower than that from the control cells, and the fumarate level was significantly increased in Flag-FH mutant overexpression cells consistently indicating the enzyme activity of mutated FH is impaired which could even affect the endogenous fumarase (Figure 2C–E).

It was known that the reduced enzyme activity might be due to the impaired binding with its substrate or the loss of its enzyme activity sites. Considering that each monomer of fumarase consists of three domains termed as D1 (residues:49–188), D2 (residues: 189–439) and D3 (residues: 440–510), and D2 functions in tetramerization, whereas D1 and D3 domains form the entrance to the independent four fumarase active sites [13,14], both the substrate binding and enzyme activity sites formation of fumarase are largely depending on the formation of the FH tetramer. Our native gel electrophoresis results showed that the fumarase mutant could not form the homotetramer compared to the fumarase wildtype suggesting the impaired tetramer formation might be the cause of the reduced enzyme activity (Figure 3A). Surprisingly, the immunofluorescence study revealed that the overexpressed FH mutant exhibited puncta structures compared with the evenly expressed FH wildtype in the cytoplasm indicating that the altered amino acid might result in the dysfunctional proteins which were accumulated in the cytoplasm to reduce its cytotoxicity (Figure 3B).

FH-deficient uterine leiomyomas tumors undergo the impairment of oxidative phosphorylation and metabolic shift to aerobic glycolysis and the increase of HIF-1α, contributing to the oncogenic growth of FH-deficient cells [28,29]. Our data showed that the cells stable overexpressing of FH mutant exhibited significantly increased growth compared to wildtype (Figure 4A). Moreover, the ECAR value of FH mutant overexpression cells was significantly higher than that from the FH wildtype overexpressed cells indicating a metabolic shift to aerobic glycolysis (Figure 4B). Further, HIF-1α was detected to be upregulated in FH mutant overexpression cells under MG132-mediated proteasomal blocked condition (Figure 4C). Overall, the *FH* mutation (c.557G>A) overexpressed cells exhibited similar cellular phenotypes as FH-deficient uterine leiomyomas cells.

The mTOR pathway was considered to play a central role in cell growth and metabolism regulating in renal cell carcinomas oncogenesis and the activation of mTOR results in the downregulation of fumarase and fumarate accumulation in Tsc1-deficient animals and cells [24,30]. Interestingly, we found that the dysfunction of fumarase could activate mTOR in a converse way. Western blotting revealed that the phospho-mTOR was significantly increased in FH mutant overexpression cells compared with the wildtype (Figure 4D). Further, as the downstream of the mTOR pathway, autophagy would be inhibited upon the activation of mTOR [26]. Consistently, our data showed that the autophagic marker LC3-II was downregulated and p62, which is a substrate of autophagy, was remarkably upregulated indicating autophagy is inhibited upon the *FH* mutation (Figure 4E). Thus, it could be suggested that the mechanism of *FH* mutation c.557G>A underlies uterine leiomyomas was due to the impaired mTOR signaling and autophagy.

## 4. Materials and Methods

### 4.1. Ethical Approval and Informed Consent

All procedures followed were in accordance with the ethical standards of the responsible committee on human experimentation (institutional and national) and with the Helsinki Declaration of 1975, as revised in 2000. Informed consent was obtained from all patients for being included in the study. The Institutional Review Board approval: SXULL2020003.

### 4.2. Subjects

A 29-year-old female patient with uterine leiomyomas (multiple) from Datong, Shanxi Province, China, was recruited 48 days after her second myomectomy which was two years after the first myomectomy. The patient was subjected to clinical and physical examinations and all the medical records were reviewed and evaluated.

### 4.3. DNA Sequencing

Whole-exome Sequencing was performed by Veritas genetics (Hangzhou, China). Whole-exome enrichment was performed using SureSelect XT Target Enrichment System (51 Mb) according to the manufacturer’s protocols (Agilent, Santa Clara, CA, USA). Captured libraries were loaded onto the HiSeq 2500 platform (Illumina, San Diego, CA, USA). An average sequencing depth of 100-fold was achieved. Paired-end sequences were first aligned to the NCBI human reference genome (hg19), and the reads were mapped by Burrows-Wheeler Alignment (BWA) v0.7.12. To identify potential mutations, we performed local realignments using the Genome Analysis Toolkit (GATK). Variants were functionally annotated and filtered using our cloud-based rare disease NGS analysis platform, based on the Ensembl (GRCh37/hg19), dbSNP, EVS, 1000 genome, ExAC and GnomAD databases. Exonic sequence alterations and intronic variants at exon-intron boundaries, with unknown frequency or minor allele frequency (MAF) < 1% and not present in the homozygous state in those databases were retained. Filtering was performed for variants in genes associated with uterine leiomyomas. Identified variants were validated and segregation analysis was performed using standard Sanger sequencing. The following primer pairs for *FH* exon 5 were used (Fw: 5′-actcacctatgacatgccac-3′, Rv:5′-cgagcagtctgactgaatct-3′).

### 4.4. Plasmid, Site-Direct Mutagenesis and Stable Cell Lines Construction

The full-length *FH* gene was purchased from Sino Biological Inc. (Beijing, China) and subcloned into the GFP-tagged and Flag-tagged lentiviral vector. The QuickChange Site-directed mutagenesis kit (Stratagene, La Jolla, CA, USA) described as previously [31] was used to generate the *FH* mutant plasmids with the primers (*FH* c.G557A-Fw: 5′-CATGTTAATAAAAGCCAGAACTCAAATGATACTTTTCCC-3′ and *FH* c.G557A-Rv: 5′-GGGAAAAGTATCATTTGAGTTCTGGCTTTTATTAACATG-3′). The pMD2.G and psPAX2 packaging plasmids were co-transfected with either Flag-FH-WT or Flag-FH-MT into HEK293T cells for producing of lentiviral particles, respectively. The HEK293T cell lines were used as target cells infected with viral supernatants and selected by puromycin.

### 4.5. Bioinformatics Analysis

Several *in silico* tools including Mutation Taster (https://www.mutationtaster.org/, accessed on 1 January 2022), PolyPhen-2 (http://genetics.bwh.harvard.edu/pph2/, accessed on 1 January 2022), PROVEAN (http://provean.jcvi.org/index.php, accessed on 1 January 2022) have been applied for the pathogenicity prediction. The evolutionary conservation of altered amino acid residue was compared across different species. The crystal structure of FH (SMTL ID: 5upp.1) [14] was used as a template to build the structural model of the FH mutant by Swiss-Model (http://swiss model.expasy.org, accessed on 1 January 2022) and the effect of the altered region was mimicked and viewed by PDB-Viewer software (SPDBV_4.10, the Swiss Institute of Bioinformatics, Basel, Switzerland).

### 4.6. Transient Transfection and Immunofluorescence

HEK293T cells were transiently transfected with pEGFP-C3, pEGFP-FH-WT or pEGFP-FH-MT by the polyetherimide (PEI, Polysciences, Inc. Warrington, PA, USA), respectively. After 24–48 h post-transfection, cells on the coverslip were fixed in 4% paraformaldehyde followed by permeabilization with Triton X-100 (0.5%) for DAPI (Merck KGaA, Darmstadt, Germany) staining and mounted in gelvatol. Images were captured as previously [32].

### 4.7. Western Blotting Analysis

Stable overexpression cells were grown up to 90% confluency in 6 well plates and lysed in RIPA buffer with 1 mM DTT, 1 mM benzamidine, 1 mM PMSF and 1× protease inhibitor cocktail. Cell resuspensions were incubated for 15 min on ice, followed by short sonication. The supernatants of lysates were collected after centrifugation at 10,000 rpm for 10 min at 4 °C. The samples were heated with 1× SDS loading buffer at 98 °C for 5 to 10 min. Following 10% or 15% SDS-PAGE and wet blot transfer, the blots were probed with one of the following primary antibodies in TBST: monoclonal anti-FLAG^®^ M2 antibody (Merck KGaA, Cat. No. F1804, Darmstadt, Germany), mTOR (phosphor-S2448) polyclonal antibody (Bioworld, Cat. No. BS4706, Nanjing, China), mTOR (S2442) polyclonal antibody (Bioworld, Cat. No. BS3611, Nanjing, China), anti-HIF-1α (Sangon, Cat. No. D162108, Shanghai, China); anti-LC3 (abcam, Cat. No. ab51520, Cambridge, UK); anti-p62 (abcam, Cat. No. ab56416, Cambridge, UK); anti-GFP (Proteintech, Cat. No. 66002-1-Ig, Wuhan, China), anti-GAPDH (Proteintech, Cat. No.60004-1-Ig, Wuhan, China). 

### 4.8. Cell Proliferation Assay

The proliferation of HEK293T cells with stable overexpression of Flag-FH-WT or Flag-FH-MT was measured by Cell Counting Kit-8 (CCK-8, YESEN, Shanghai, China). Briefly, 2000 cells per well were seeded in a 96-well plate and the cell numbers were detected every two days according to the manufacturer’s instructions. The OD values representing the cell numbers were read at 450 nm.

### 4.9. FH activity Measurement and Fumarate Quantification

Fumarase Activity Colorimetric Assay Kit (MAK206; Merck) and Fumarate Assay Kit (MAK060; Merck) were used. Briefly, 10^6^ HEK293T cells with stable overexpression of Flag, Flag-FH-WT or Flag-FH-MT were lysed with 100 μL ice-cold fumarase assay buffer or fumarate buffer from the corresponding kit. The supernatants were collected after centrifugation at 13,000× *g* for 10 min. Reaction mixes were prepared and incubated following the manufacturer’s instructions. The absorbances were measured immediately at 450 nm using a spectrum microplate reader (Synergy H1, Biotek).

### 4.10. Metabolic Measurements in Cultured Cells

The extracellular acidification rate (ECAR) was measured in Flag, Flag-FH-WT or Flag-FH-MT stable overexpression HEK293T cells using Seahorse XFe24 Flux Analyzer (Agilent, Santa Clara, CA, USA). Briefly, 5 × 10^5^ HEK293T cells/well were seeded in XF 24-well cell culture microplate with 200 μL culture medium and incubated for 24 h at 37 °C in 5% CO_2_. Cells were incubated for 1 h in a non-CO_2_ incubator after 200 μL basic medium (pH 7.4) replacement. ECAR was detected post-additions of 10 mM glucose, 1 μM oligomycin and 50 mM 2-DG. The mix-wait-measure time program was selected as 3 min-2 min-3 min.

### 4.11. Native Gel Electrophoresis

The transiently transfected or stable overexpression with FH wildtype or FH mutant HEK293T cells were lysed in non-denatured lysis buffer (50 mM Tris/HCl, pH8.0; 150 mM NaCl; 1% NP-40). After ice-cold incubation and short sonication, the lysates were cleared by centrifugation at 10,000 rpm for 10 min at 4 °C. The supernatants were mixed with 5× non-denature loading buffer followed by 6% native gel electrophoresis.

### 4.12. Statistical Analysis

All data are presented as the mean ± SD from at least three separate experiments. The statistical significance of differences between FH wildtype and mutant was determined using the GraphPad Prism software 8 (GraphPad Software, Inc., La Jolla, CA, USA). *p* < 0.05 was considered as being significant.

## 5. Conclusions

Our study reports the *FH* missense mutation c.557G>A results in the uterine leiomyomas phenotype. Functional studies confirm the pathogenicity of this mutation, and the probable molecular mechanism predisposes to the disease (Figure 4F). In the presence of FH wildtype, fumarase homotetramer is formed to exhibit the enzyme activity, resulting in the inhibition of mTOR signaling. However, in the presence of the FH mutant, fumarase homotetramer is impaired and the enzyme activity is lost, resulting in the activation of mTOR, which further leads to the upregulation of HIF-1α and downregulation of autophagy (Figure 4F). Data in this study will expand the uterine leiomyomas mutation database and provide new insight into the molecular mechanism and clinical diagnosis of the disease.

## Data Availability

All primary data are available upon reasonable request.
